# Changes in Gene Expression Profiling and Phenotype in Aged Multidrug Resistance Protein 4-Deficient Mouse Retinas

**DOI:** 10.3390/antiox10030455

**Published:** 2021-03-15

**Authors:** Kyung Woo Kim, Sentaro Kusuhara, Atsuko Katsuyama-Yoshikawa, Sho Nobuyoshi, Megumi Kitamura, Sotaro Mori, Noriyuki Sotani, Kaori Ueda, Wataru Matsumiya, Akiko Miki, Takuji Kurimoto, Hisanori Imai, Makoto Nakamura

**Affiliations:** Division of Ophthalmology, Department of Surgery, Kobe University Graduate School of Medicine, 7-5-1 Kusunoki-cho, Chuo-ku, Kobe 650-0017, Japan; kim6048@med.kobe-u.ac.jp (K.W.K.); monpey_696sp@yahoo.co.jp (A.K.-Y.); rainbow_milkyway_wowwow@yahoo.co.jp (S.N.); megkita@med.kobe-u.ac.jp (M.K.); smori@med.kobe-u.ac.jp (S.M.); sn1117@med.kobe-u.ac.jp (N.S.); kueda@med.kobe-u.ac.jp (K.U.); ytkmatsu@med.kobe-u.ac.jp (W.M.); acacyey@med.kobe-u.ac.jp (A.M.); kuri1201@med.kobe-u.ac.jp (T.K.); hisimai@med.kobe-u.ac.jp (H.I.); manakamu@med.kobe-u.ac.jp (M.N.)

**Keywords:** multidrug resistance protein 4, ATP-binding cassette (ABC) transporters, aging, retina, mouse, electroretinogram

## Abstract

Multidrug resistance protein 4 (MRP4) is an energy-dependent membrane transporter responsible for cellular efflux of a broad range of xenobiotics and physiological substrates. In this trial, we aimed to investigate the coeffects of aging and MRP4 deficiency using gene expression microarray and morphological and electrophysiological analyses of mouse retinas. *Mrp4*-knockout (null) mice and wild-type (WT) mice were reared in the same conditions to 8–12 weeks (young) or 45–55 weeks (aged). Microarray analysis identified 186 differently expressed genes from the retinas of aged *Mrp4*-null mice as compared to aged WT mice, and subsequent gene ontology and KEGG pathway analyses showed that differently expressed genes were related to lens, eye development, vision and transcellular barrier functions that are involved in metabolic pathways or viral infection pathways. No significant change in thickness was observed for each retinal layer among young/aged WT mice and young/aged *Mrp4*-null mice. Moreover, immunohistochemical analyses of retinal cell type did not exhibit an overt change in the cellular morphology or distribution among the four age/genotype groups, and the electroretinogram responses showed no significant differences in the amplitude or the latency between aged WT mice and aged *Mrp4*-null mice. Aging would be an insufficient stress to cause some damage to the retina in the presence of MRP4 deficiency.

## 1. Introduction

In general, aging affects all organ systems in individuals via several physiological and pathological processes [1]. Oxidative stress is a major modifier of organ dysfunction and has been linked to age-related diseases, such as cardiovascular diseases, diabetes, chronic kidney diseases and neurodegenerative diseases [2,3,4,5]. A recent systematic review suggests the association of a long lifespan with less oxidative damage, although the authors admitted that the complexity of the etiology and the effects of gene–environment interaction occurring throughout the lifetime remain unclear [6]. Research has shown that oxidative stress regulates the expression of a subset of efflux ATP-binding cassette (ABC) transporters that are located on the cell membrane that protects cells against oxidative stress, including P-glycoprotein, multidrug resistance-related proteins and breast cancer-related proteins [7]. The retina is one of the most susceptible tissues to oxidative damage because it has the highest oxygen consumption rate per kg of the body among body organs, thereby leading to exposure to a large amount of endogenous reactive oxygen species [8]. It is well known that oxidative stress leads to various types of ocular disorders, including retinal dystrophy [9,10]. For example, *ABCA4* is the gene coding ABC transporter (subfamily A, member 4). Its variants are associated with retinal dystrophies such as autosomal recessive Stargardt disease, and recent studies are unraveling complexities in the genetics of *ABCA4*-associated retinopathy [11,12].

Multidrug resistance protein 4 (MRP4) belongs to the C subfamily of ABC transporters and maintains a cellular environment by functioning as an efflux pump. MRP4 transports a wide variety of endogenous and xenobiotic compounds out of cells, some of which usually induce oxidative stress [7,13]. Previous loss-of-function studies imply that mouse MRP4 does not play a critical physiological role because *Mrp4*-deficient mice reared under normal conditions are viable and do not show any overt organ dysfunction [8,14,15,16]. However, it is known that *Mrp4*-deficient mammals express abnormal reactions to various types of stress, such as carotid artery injury, hypoxia-induced pulmonary arterial hypertension and systemic administration of forskolin, an adenylyl cyclase activator [17,18,19]. In the relationship between aging and oxidative stress, MRP4 may play a role in the process of aging. An experimental study that comprehensively investigated mRNA profiles for drug-metabolizing enzymes and transporters showed that mRNA levels of Mrp4 in the liver were higher in aged mice [20]. In another in vivo study, MRP4 was overexpressed in the hearts of aged rats, and pharmacological inhibition of MRP4 restored the positive inotropic effect of β-adrenoreceptor stimulation that was reduced in the senescent heart [21]. Moreover, in *Drosophila*, loss-of-function mutations of *dMRP4*, a *Drosophila* homolog of human *MRP4*, increased sensitivity to oxidative stress and reduced lifespan [22].

Recently, MRP4 has been recognized as an attractive pharmacological target because it contributes not only to detoxification but also to the homeostasis of several signaling molecules. Previous xenobiotic studies identified FDA-approved drugs or bioactive compounds with inhibition potency for MRP4. FDA-approved drugs with MRP4-inhibitory function include drugs that are administered commonly in clinical practice, such as nonsteroidal anti-inflammatory drugs, hypolipidemic agents, antithrombotic agents and angiotensin-converting enzyme inhibitors [13,23,24]. Therefore, aged individuals with systemic diseases might have been exposed to one of these drugs for years. The retina, used as a window to the brain, is a part of the central nervous system (CNS), and animal experiments have confirmed that MRP4 is expressed predominantly on vascular endothelial cells in the retina, as in the brain [13,25,26].

Although evidence is accumulating on the roles of MRP4 in physiological and pathological conditions, the impact of the loss of MRP4 on aging remains elusive. Therefore, to obtain insights into the coeffects of MRP4 deficiency and aging on the CNS, we investigated the changes in gene expression profiles and phenotype in aged mouse retinas; further, we compared these parameters between wild-type (WT) and *Mrp4*-null mice.

## 2. Materials and Methods

### 2.1. Mice

All the animal procedures were reviewed and approved by Kobe University Animal Care and Use Committee (permission numbers P141204 and P200406). The study was performed as per Kobe University animal experimentation regulations and the Association for Research in Vision and Ophthalmology (ARVO) Statement for the Use of Animals in Ophthalmic and Vision Research. Mrp4-knockout mice (gifted from Dr. John D. Schuetz at St. Jude Children’s Research Hospital, Memphis, TN, USA) with a C57BL/6J genetic background were maintained and used. All of the mice were maintained under standard laboratory conditions (12/12 h light/dark cycle and 20–24 °C; food and water were provided without restriction). For all the procedures, anesthetic effect was achieved by intraperitoneal injection of a mixture of 0.75 mg/kg medetomidine, 4.0 mg/kg midazolam and 5.0 mg/kg butorphanol.

### 2.2. Gene Expression Microarray

Retinal tissues were harvested from three mice aged 45–55 weeks and stored in an Eppendorf tube filled with TRIzol (Thermo Fisher Scientific, Waltham, MA, USA) at −80 °C until RNA extraction. We prepared four samples for each mouse genotype (WT and *Mrp4^−/−^*). RNA extraction and the following RNA microarray and KEGG pathway analysis were performed by Macrogen Inc. (Tokyo, Japan). One-color Cy3 RNA labeling and array hybridization to Agilent SurePrint G3 Mouse Gene Expression Arrays 8 × 60 K Ver. 2.0 (Agilent Technologies, Santa Clara, CA, USA) were performed. In brief, labeled cRNA was prepared from 1–5 µg total RNA using Agilent’s Quick Amp Labeling Kit. Following fragmentation, 1.65 µg of cRNA was hybridized to the Agilent expression microarray as per the protocols recommended by the manufacturer. Then the arrays were scanned using Agilent Technologies’ G4900DA SG12494263, and array data export processing and analysis were performed with Agilent Feature Extraction v11.0.1.1. The raw data for the same gene were then summarized automatically in the Agilent Feature Extraction protocol to generate a raw data text file, providing expression data for each gene that was probed on the array.

### 2.3. Data Analyses

Array probes that had Flag A in the samples were filtered out. Selected gProcessed Signal value was transformed by logarithm and normalized using the quantile method. Statistical significance of the expression data was determined using fold change and the local pooled error (LPE) test, wherein the null hypothesis was that no difference exists between the two groups. Hierarchical cluster analysis was performed using complete linkage and Euclidean distance as a measure of similarity. Significant counting by the absolute value of fold change ≥1.5 and *p*-value LPE < 0.05 was performed using the processed data. Then, Gene-Enrichment and Functional Annotation analysis for the significant probe list was performed using Gene Ontology (GO) (http://geneontology.org, accessed on 28 November 2020) and KEGG (http://kegg.jp, accessed on 28 November 2020). All data analysis and visualization of differentially expressed genes were performed using R 3.3.3 (www.r-project.org, accessed on 28 November 2020). Primary microarray data after significant counting have been deposited in NCBI’s Gene Expression Omnibus (GEO) and are accessible through GEO series accession number GSE162376.

### 2.4. Immunohistochemistry and H&E Staining

Retinal whole-amount immunostaining, immunohistochemical staining of tissue sections and H&E staining for frozen retinal sections were performed as described previously [18,26,27]. Acquisition and analyses of fluorescence images were performed using a confocal laser scanning microscope (LSM-700; Carl Zeiss, Tokyo, Japan), and H&E retinal sections were photographed with BZ-8000 (Keyence, Osaka, Japan). The primary and secondary antibodies used were rat anti-CD31 antibody (BD Pharmingen, San Diego, CA, USA), Cy3-conjugated mouse anti-glial fibrillary acidic protein (GFAP) antibody (Sigma-Aldrich), rabbit anti-glutamine synthetase antibody (Thermo Fisher Scientific, Waltham, MA, USA), rabbit anti-calbindin D-28K (Merck Millipore, Burlington, MA, USA), sheep anti-Chx10 antibody (Exalpha Biological, Shirley, MA, USA), rhodamine-conjugated peanut agglutinin (Vector Laboratories, Burlingame, CA, USA), Cy3-conjugated donkey anti-anti-rat IgG (H + L) antibody (Jackson ImmunoResearch, West Grove, PA, USA), Alexa Fluor 488 conjugated donkey anti-rat IgG antibody (Invitrogen), Alexa Fluor 488 conjugated donkey anti-rabbit IgG antibody (Invitrogen), Alexa Fluor 488 conjugated donkey anti-sheep IgG antibody (Invitrogen) and TO-PRO-3 (Invitrogen). We used Image J software for quantitative analyses (AngioTool software for the analyses of vessel area and total vessel length) [28]. The thickness of each retinal layer was quantified by manually measuring the retinal layers at 100 µm from the optic disc on the H&E retinal section.

### 2.5. Electroretinography Recording

Electroretinography (ERG) recording was conducted as reported previously [29,30], with some modifications. All the animals were dark-adapted overnight before ERG recordings, and all the procedures were performed under dim red light. Mice were anesthetized and positioned on a built-in heating pad that maintains the body temperature at 37 °C. After dilating the pupils using 2.5% phenylephrine and 1.0% tropicamide eye drop, (Santen Pharmaceutical Co., Osaka, Japan), a contact lens electrode embedded with gold wire was placed on the cornea as an active electrode (Mayo, Aichi, Japan), and a chloride silver plate was placed in the mouth as a reference electrode. The ERGs were recorded using commercially available equipment with a Ganzfeld bowl (Mayo, Aichi, Japan). Scotopic recordings were obtained from dark-adapted animals at the following increasing light intensities. Responses were amplified 10,000 times and bandpass-filtered from 0.3 to 500 Hz (PuREC PC-100, Mayo). For the recording of scotopic threshold response (STR), serially increasing luminescence intensities of −6.1, −5.5, −5.1, −4.6 and −4.1 log sc td s were used. Responses were amplified differentially and bandpass-filtered at 0.125–50 Hz, and responses from 80 repeated stimuli for each intensity were averaged. Photopic recordings were performed after 5-min light adaptation intervals on a background light intensity of 1.5 log sc td. Responses were amplified differentially and bandpass-filtered at 0.3–300 Hz, and the responses from 30 (20 for flicker) repeated stimuli for each intensity were averaged.

### 2.6. Statistical Analyses

Statistical analyses were performed using MedCalc software version 11.3 (MedCalc Software, Mariakerke, Belgium). All the descriptive statistics are presented as mean ± standard deviation values (all the experiments were performed at least in triplicate). Comparisons between the groups and among >3 groups were analyzed using Student’s t-test and analysis of variance (ANOVA), respectively. The Student–Newman–Keuls test for all pairwise comparisons was conducted when the F ratio was significant in ANOVA. A *p*-value <0.05 was considered to indicate statistical significance. Microarray data analyses were conducted as described above.

## 3. Results

### 3.1. Altered Gene Expression Profile in Aged Mrp4-Null Mouse Retinas

To examine comprehensive transcriptome information on aged *Mrp4*-null mouse retinas, we performed oligonucleotide-based microarray analysis with Agilent SurePrint G3 Mouse Gene Expression Arrays 8 × 60 K Ver. 2.0 that covers content from RefSeq, Ensembl, RIKEN, UniGene and GenBank databases to provide full coverage of the mouse transcriptome: 27,112 Entrez Genes, 4578 lncRNAs, 39,430 Entrez Gene RNAs and 16,251 lincRNAs. As compared to age-matched WT mice, 186 differently expressed genes were identified from the retinas of aged (about 50 weeks old) *Mrp4*-null mice with the absolute value of fold change ≥ 1.5 and *p*-value of LPE < 0.05 (Table S1). The top 10 up- or downregulated genes are listed in Table 1.

**Figure 1:**
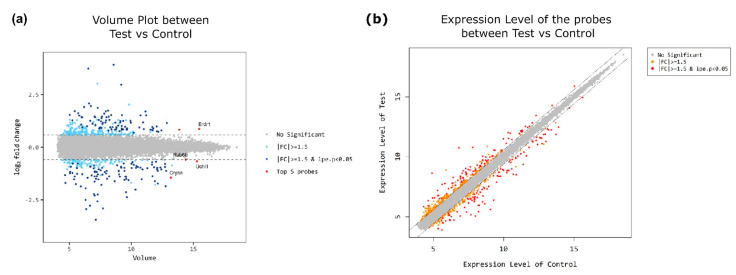
Volume and scatter plots of the normalized microarray data. (**a**) Volume plot. (**b**) Scatter plot. Test, Mrp4-null mice; control, wild-type mice; fc, fold change; lpe, local pooled error.

**Figure 2:**
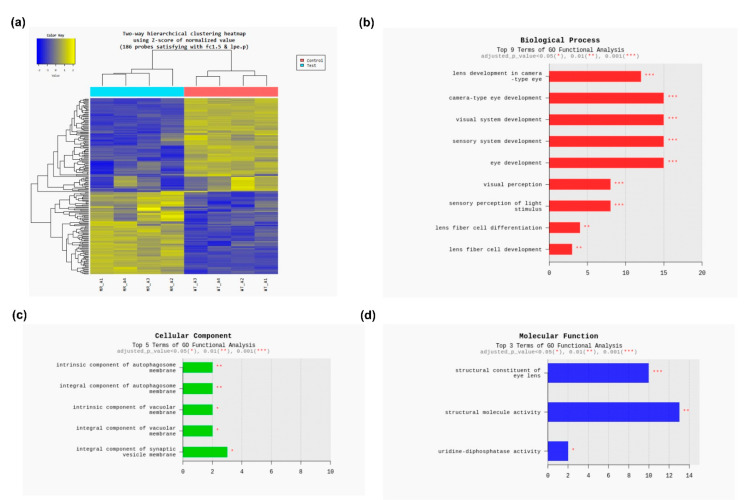
Microarray data analyses. (**a**) Cluster analysis. (**b**) GO analysis (biological process). (**c**) GO analysis (cellular component). (**d**) GO analysis (molecular function). Test, *Mrp4*-null mice; control, wild-type mice; MR, *Mrp4*-null mice; WT, wild-type mice; fc, fold change; lpe, local pooled error; GO, gene ontology.

Figure 1 and Figure 2 present the clustering of the gene expression data and scatter and volume plots of the microarray analysis. GO analysis results showed that differentially expressed genes were significantly enriched in biological processes, molecular functions and cell components: lens development in the camera-type eye, camera-type eye development, visual system development, sensory system development, eye development, sensory organ development, visual perception, sensory perception of light stimulus, lens fiber cell differentiation, lens fiber cell development, structural constituent of eye lens, structural molecule activity, uridine-diphosphatase activity, intrinsic component of autophagosome membrane, integral component of autophagosome membrane, intrinsic component of vacuolar membrane, integral component of vacuolar membrane and integral component of synaptic vesicle membrane (Figure 2b–d). Subsequent KEGG pathway analysis revealed that differently expressed genes were mainly involved in the “metabolic pathway”, “glycerophospholipid metabolism”, “herpes simplex virus 1 infection” and “Kaposi sarcoma-associated herpes virus infection” (Figure S1).

### 3.2. Thickness Changes in Each Retinal Layer

**Figure 3:**
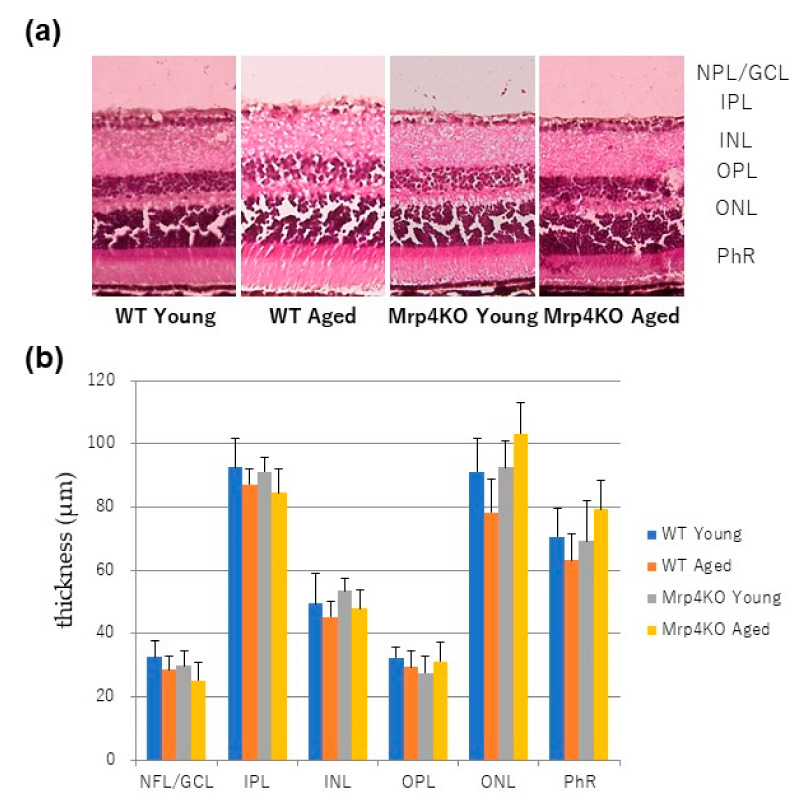
H&E staining results. (**a**) Representative retinal section images of H&E staining. (**b**) Quantitative comparison of thickness in each retinal layer. No significant difference was observed (*n* = 6). WT, wild-type mice; Mrp4KO, *Mrp4*-null mice; NFL, nerve fiber layer; GCL, ganglion cell layer; IPL, inner plexiform layer; INL, inner nuclear layer; OPL, outer plexiform layer; ONL, outer nuclear layer; PhR, photoreceptor layer.

We hypothesized that differently expressed genes in aged *Mrp4*-null mouse retinas have some associations with the retinal architecture. Therefore, we then assessed the thickness changes in each retinal layer: nerve fiber layer (NFL)/ganglion cell layer (GCL) complex, inner plexiform layer (IPL), inner nuclear layer (INL), outer plexiform layer (OPL), outer nuclear layer (ONL) and photoreceptor layer (PhR). Figure 3 shows the results of H&E staining. No significant change in the thickness was observed for each retinal layer among young (8–12 weeks) WT mice, aged (45–55 weeks) WT mice, young *Mrp4*-null mice and aged *Mrp4*-null mice.

### 3.3. Morphology and Distribution of Each Retinal Cell Type

**Figure 4:**
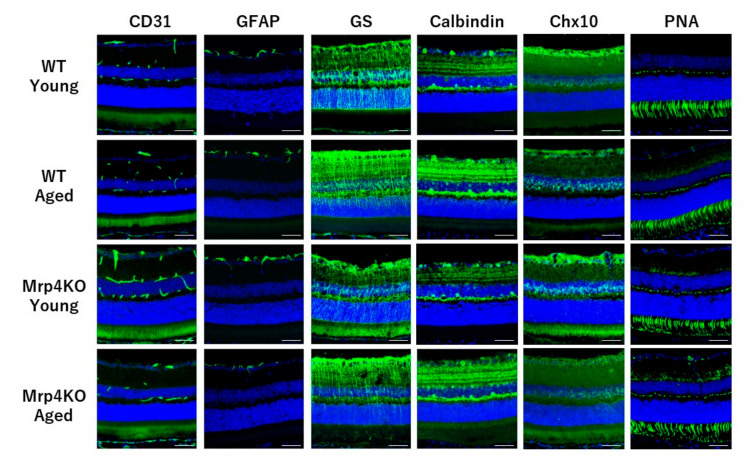
Immunohistochemical staining results of the retinal section. Bar = 100 μm. WT, wild-type mice; Mrp4KO, Mrp4-null mice; GFAP, glial fibrillary acidic protein; GS, glutamine synthetase; PNA, peanut agglutinin.

In response to the results of H&E staining, we checked the morphology and distribution of each retinal cell type because the changes related to aging and *Mrp4* deficiency might be detected at the cellular level. Section immunohistochemical staining demonstrated no apparent changes in the morphology and distribution among young/aged WT mice and young/aged *Mrp4*-null mice for vascular endothelial cells (CD31), astrocytes (GFAP), Muller cells (glutamine synthetase), horizontal and amacrine cells (calbindin), bipolar cells (Chx10) or photoreceptors (peanut agglutinin) (Figure 4).

**Figure 5:**
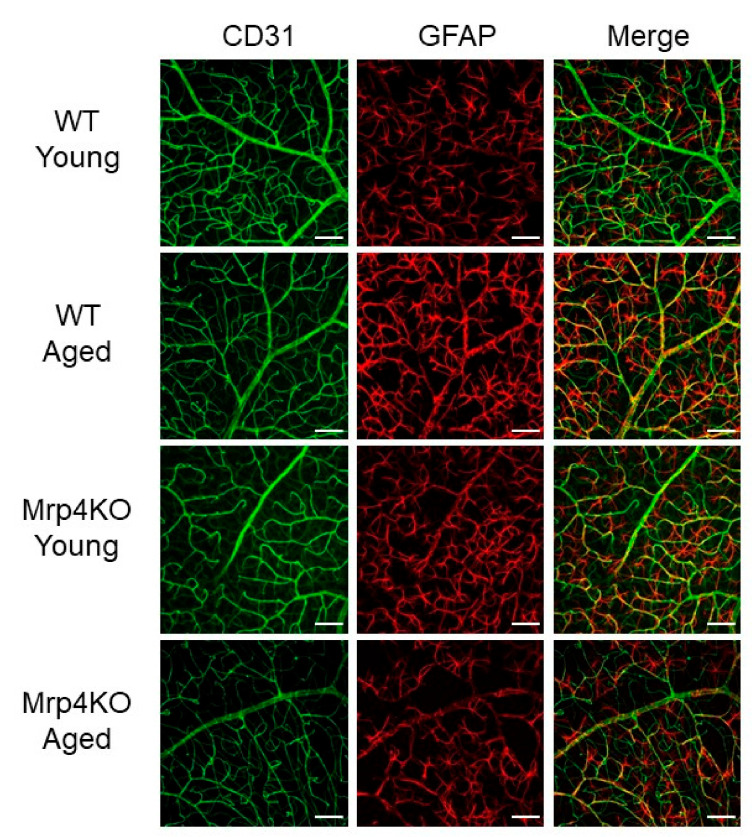
Immunohistochemical staining results of whole mount retina. Retinal vasculature and astrocyte network at the superficial layer of the retina. Bar = 100 μm.

**Figure 6:**
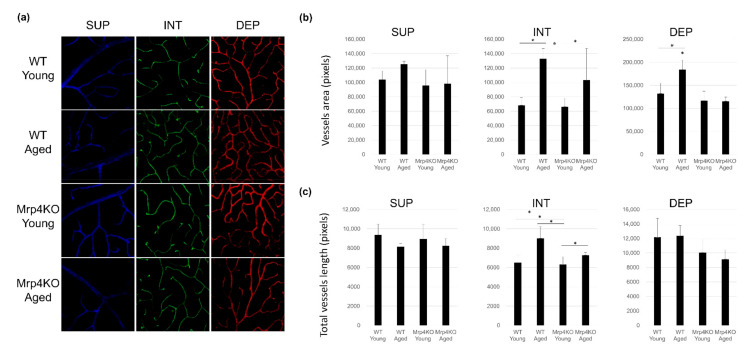
Retinal vasculature at the different levels of the retinal layer. (**a**) Representative images (x20 objective). (**b**) Quantitative comparison of vessel area in each retinal layer (*n* = 3–4). (**c**) Quantitative comparison of the total vessel length in each retinal layer (*n* = 3–4). WT, wild-type mice; Mrp4KO, Mrp4-null mice; GFAP, glial fibrillary acidic protein; SUP, superficial retinal layer; INT, intermediate retinal layer; DEP, deep retinal layer. * *p* < 0.05.

Retinal whole-mount immunostaining was performed for the two-dimensional evaluation of retinal vessels and the astrocyte network that could not be used for performing section immunohistochemistry. Further, the results showed no overt change in young/aged WT mice and young/aged *Mrp4*-null mice in terms of the retinal vasculature or astrocyte network, although some layers showed a small but significant difference in the vascular area, total vascular length or both (Figure 5 and Figure 6).

### 3.4. Electrophysiological Function of the Retina

**Figure 7:**
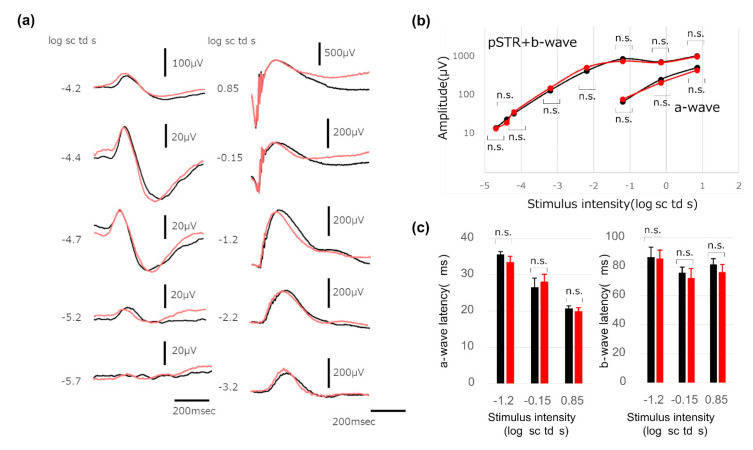
Results of scotopic electroretinography (ERG) phenotype. (**a**) Representative dark-adapted ERG recordings. (**b**) Quantitative analyses of the amplitude of pSTR + b-wave (*n* = 6). (**c**) Quantitative analyses of the latency of a- and b-waves (*n* = 6). Error bar = standard deviation. Black lines or black bars represent the data from aged (45 weeks old) WT mice and red lines or red bars represent the data from aged (45 weeks old) *Mrp4*-null mice. pSTR, positive scotopic threshold response; n.s., not significant.

In order to test whether the retinas of aged *Mrp4*-null mice have the same retinal function as those of WT mice, we conducted electrophysiological analyses. Electroretinography (ERG) performed for aged *Mrp4*-null mice showed no significant difference from aged WT mice in terms of the amplitude and latency of a- and b-waves and the amplitude of positive STR (Figure 7 and Figure S2).

## 4. Discussion

In the current study, we performed a comprehensive transcriptome analysis and identified 186 differently expressed genes in aged *Mrp4*-null mice as compared to aged WT mice and obtained the information on significant GO terms and KEGG pathways using the differently expressed gene. However, no overt retinal phenotype related to aging or *Mrp4* deficiency was observed in the retinal architecture, morphology and distribution of each retinal cell type or electrophysiological function.

A total of 186 differently expressed genes would reflect the effects of blood–retinal barrier (BRB) dysfunction on the neural retina because (1) MRP4 is almost exclusively expressed in vascular endothelial cells in the retina [25,26], (2) endothelial cells are major components of the BRB [31,32], (3) MRP4 restricts transcellular transport [23] and (4) endothelial cells account for only a small cell population (0.1%) in the retina [33]. The GO terms significantly classified using 186 differently expressed genes involved those related to the lens (lens development in camera-type eye, lens fiber cell differentiation and lens fiber cell development), eye development (camera-type eye development, visual system development, sensory system development, eye development and sensory organ development) and vision (visual perception and sensory perception of light stimulus) for biological process. We confirmed that the extracted GO terms in our study varied from those in other studies that investigated the effect of ocular inflammation, ultraviolet radiation or strain [34,35,36]. It surprised us that differently expressed genes were enriched within lens-related GO because the retina and lens are recognized as two entirely different tissue types. However, this result might convey an important message to us. For example, crystallins are primarily found as major structural proteins of the ocular lens; however, has been disclosed that αB-crystallin acts as a regulator of retinal angiogenesis [37]. The significant GO terms for molecular function or cellular components, such as structural molecule activity, intrinsic component of vacuolar membrane and integral component of synaptic vesicle membrane might be associated with the MRP4 transcellular barrier function in the CNS [38]. Specific signaling pathways affected by *Mrp4* deficiency in aged mouse retinas included “metabolic pathway”, “glycerophospholipid metabolism”, “herpes simplex virus 1 infection” and “Kaposi sarcoma-associated herpes virus infection”. Although distinct relationships between the first two pathways and MRP4 remain unknown, the last two pathways might be associated with MRP4 function as an efflux transporter because antiviral agents are MRP4 substrates [23,39].

Before focusing on some key genes that were significantly upregulated or downregulated in aged *Mrp4*-null mice in the list, we checked the severity of the impact on the retina aging and *Mrp4* deficiency. Thickness change in the specific retinal layer is likely to be a good marker of retinal degeneration [40]; therefore, we measured the thickness of retinal layers in young/aged WT mice and young/aged *Mrp4*-null mice and performed a comparison. Unexpectedly, although >100 of the genes are differently expressed in aged *Mrp4*-null mouse retinas, changes in the thickness of each retinal layer did not show any significant differences. We checked some retinal sections from WT and *Mrp4*-null mice aged 2 years and found no overt retinal phenotype (data not shown). Moreover, immunohistochemical analyses of the retinal cell type did not exhibit any obvious change in the cellular morphology or distribution among the four age/genotype mouse groups. Inconspicuous vascular phenotypes observed in the intermediate or deep retinal layer, or in both layers, might be attributed to *Mrp4* deficiency, aging or both, because we previously disclosed that *Mrp4* deficiency led to retinal vascular phenotypes after forskolin administration in neonatal mice [18]. An extensive electrophysiological analysis confirmed these histological results, showing no significant ERG responses in the amplitude or the latency between the aged WT mice and aged *Mrp4*-null mice. Based on these experimental results, we concluded that aging is an insufficient stress to cause some damage to the retina in *Mrp4*-null mice. However, we are currently unable to explain why aged *Mrp4*-null mice show no evident retinal phenotype in spite of the presence of many differently expressed genes in the retina. We made the following speculations: (1) as the mice had been housed under well-controlled and specific-pathogen-free conditions, accumulated endogenous and exogenous stresses were beneath the threshold that caused retinal damage; (2) other efflux transporter family proteins expressed in the retina compensated for the lack of MRP4; and (3) the pleiotropic effects of the loss of MRP4 function canceled the molecular pathways causing retinal dysfunction. In any case, the point is that the coeffects of MRP4 deficiency and aging are unlikely to cause serious damage to the retina. It might be a boon to patients who continue taking drugs with MRP4-inhibitory activity; however, further studies should be performed to find a resolution of this issue because they have systemic diseases, such as hyperlipidemia, hypertension and cancer, that may impose another stress in addition to aging [13,23,24].

This study has certain limitations. First, microarray analysis was performed at only one time point. If microarray data were acquired at many different time points, the coeffects of MRP4 and chronological aging on retinal phenotype could be further understood. Second, we used whole retinas to investigate the differences in gene expression. Although the major component of the retina is the neuron, the retina has several types of neurons as well as glial cells and vascular endothelial cells. Therefore, we observed the sum of gene expression levels derived from different cell types in our microarray data. Omics analysis of the data from isolated retinal cells would be ideal, as some investigators performed studies using cultured cells [41,42,43]. However, inconspicuous retinal phenotypes observed in aged *Mrp4*-null mice had not provoked us to conduct further experiments (e.g., RNA-seq experiments using FACS-isolated retinal cells). Third, although technically difficult, we did not evaluate cell behaviors that would facilitate brain research because the retina is an ideal tissue for in vivo imaging, and the cellular events that occurred in the retina were linked to those in the brain.

In conclusion, MRP4 deficiency in aged mice caused different gene expression than in WT mice. The analyzed gene profiles suggest that aging and MRP4 deficiency affect the expression in the retina of genes associated with the lens, eye development, vision and transcellular barrier function that are involved in metabolic pathways or viral infection pathways. However, the magnitude of the impact of gene expression changes observed in aged *Mrp4*-null mice on the retinal morphology and function would be small. Aging is insufficient as a stress to cause some damage to the retina in *Mrp4*-null mice, and further studies should be performed to investigate the coeffects of Mrp4 deficiency and other types of stress, such as hyperglycemia and inflammation.

## Figures and Tables

**Table 1:** The top 10 up- or downregulated probe list in the retina of aged *Mrp4* knockout mice. LPE, local pooled error; N.A., not applicable.

**Upregulated**				
**Probe ID**	**Gene Symbol**	**RefSeq Accession**	**Fold Change**	**LPE**
A_52_P630867	Abcc4	NM_001033336	15.131763	0
A_51_P431785	Myom2	NM_008664	13.353321	0
A_51_P151126	Cd52	NM_013706	7.874139	0
A_55_P2185504	Masp2	NM_010767	4.247988	3.44 × 10^−12^
A_51_P368009	E2f2	NM_177733	3.894574	6.01 × 10^−12^
A_51_P445153	Spry2	NM_011897	3.755293	5.16 × 10^−12^
A_30_P01028367	N.A.	N.A.	3.664921	2.52 × 10^−12^
A_51_P251402	Tgds	NM_029578	3.344442	1.20 × 10^−11^
A_51_P112966	Ch25h	NM_009890	3.289766	7.45 × 10^−3^
A_30_P01033318	N.A.	N.A.	3.262382	4.25 × 10^−11^
**Downregulated**				
**Probe ID**	**Gene Symbol**	**RefSeq Accession**	**Fold Change**	**LPE**
A_55_P2003614	4930480K23Rik	NR_130157	−10.853326	9.49 × 10^−41^
A_55_P2032445	Slc25a37	NM_026331	−7.660266	1.36 × 10^−23^
A_51_P361286	Agpat5	NM_026792	−7.499705	1.30 × 10^−29^
A_66_P133273	N.A.	N.A.	−6.848760	4.22 × 10^−25^
A_52_P70381	N.A.	N.A.	−5.933803	1.07 × 10^−21^
A_66_P131433	N.A.	N.A.	−5.677915	3.04 × 10^−18^
A_52_P529374	Enox1	NM_172813	−5.194814	2.29 × 10^−26^
A_30_P01031079	N.A.	N.A.	−4.905352	9.37 × 10^−16^
A_52_P555603	Apbb2	N.A.	−4.743147	2.78 × 10^−15^
A_55_P2737912	Lgi3	NR_130157	−4.697855	2.53 × 10^−16^

## Data Availability

According to the journal guidelines, RNA-Seq data was deposited in NCBI’s Gene Expression Omnibus and are accessible through GEO series accession number GSE162376.

## Supplementary Materials

The following are available online at https://www.mdpi.com/2076-3921/10/3/455/s1, Figure S1: KEGG pathway analysis result, Figure S2: Results of photopic electroretinogram (ERG) phenotype, Table S1: Differentially expressed gene list.

## References

[1] Da Costa J.P., Vitorino R., Silva G.M., Vogel C., Duarte A.C., Rocha-Santos T. (2016). A synopsis on aging-Theories, mechanisms and future prospects. Ageing Res. Rev..

[2] El Assar M., Angulo J., Rodriguez-Manas L. (2013). Oxidative stress and vascular inflammation in aging. Free Radic. Biol. Med..

[3] Gerber P.A., Rutter G.A. (2017). The Role of Oxidative Stress and Hypoxia in Pancreatic Beta-Cell Dysfunction in Diabetes Mellitus. Antioxid. Redox Signal..

[4] Jha J.C., Banal C., Chow B.S., Cooper M.E., Jandeleit-Dahm K. (2016). Diabetes and Kidney Disease: Role of Oxidative Stress. Antioxid. Redox Signal..

[5] Schrag M., Mueller C., Zabel M., Crofton A., Kirsch W.M., Ghribi O., Squitti R., Perry G. (2013). Oxidative stress in blood in Alzheimer’s disease and mild cognitive impairment: A meta-analysis. Neurobiol. Dis..

[6] Belenguer-Varea A., Tarazona-Santabalbina F.J., Avellana-Zaragoza J.A., Martinez-Reig M., Mas-Bargues C., Ingles M. (2020). Oxidative stress and exceptional human longevity: Systematic review. Free Radic. Biol. Med..

[7] Grewal G.K., Kukal S., Kanojia N., Saso L., Kukreti S., Kukreti R. (2017). Effect of Oxidative Stress on ABC Transporters: Contribution to Epilepsy Pharmacoresistance. Molecules.

[8] Leggas M., Adachi M., Scheffer G.L., Sun D., Wielinga P., Du G., Mercer K.E., Zhuang Y., Panetta J.C., Johnston B. (2004). Mrp4 confers resistance to topotecan and protects the brain from chemotherapy. Mol. Cell Biol..

[9] Domènech E.B., Marfany G. (2020). The Relevance of Oxidative Stress in the Pathogenesis and Therapy of Retinal Dystrophies. Antioxidants.

[10] Donato L., Scimone C., Alibrandi S., Abdalla E.M., Nabil K.M., D’Angelo R., Sidoti A. (2020). New Omics-Derived Perspectives on Retinal Dystrophies: Could Ion Channels-Encoding or Related Genes Act as Modifier of Pathological Phenotype?. Int. J. Mol. Sci..

[11] Cremers F.P.M., Lee W., Collin R.W.J., Allikmets R. (2020). Clinical spectrum, genetic complexity and therapeutic approaches for retinal disease caused by ABCA4 mutations. Prog. Retin. Eye Res..

[12] D’Angelo R., Donato L., Venza I., Scimone C., Aragona P., Sidoti A. (2017). Possible protective role of the ABCA4 gene c.1268A>G missense variant in Stargardt disease and syndromic retinitis pigmentosa in a Sicilian family: Preliminary data. Int. J. Mol. Med..

[13] Russel F.G., Koenderink J.B., Masereeuw R. (2008). Multidrug resistance protein 4 (MRP4/ABCC4): A versatile efflux transporter for drugs and signalling molecules. Trends Pharmacol. Sci..

[14] Belinsky M.G., Guo P., Lee K., Zhou F., Kotova E., Grinberg A., Westphal H., Shchaveleva I., Klein-Szanto A., Gallo J.M. (2007). Multidrug resistance protein 4 protects bone marrow, thymus, spleen, and intestine from nucleotide analogue-induced damage. Cancer Res..

[15] Lin Z.P., Zhu Y.L., Johnson D.R., Rice K.P., Nottoli T., Hains B.C., McGrath J., Waxman S.G., Sartorelli A.C. (2008). Disruption of cAMP and prostaglandin E2 transport by multidrug resistance protein 4 deficiency alters cAMP-mediated signaling and nociceptive response. Mol. Pharmacol..

[16] Mennone A., Soroka C.J., Cai S.Y., Harry K., Adachi M., Hagey L., Schuetz J.D., Boyer J.L. (2006). Mrp4-/- mice have an impaired cytoprotective response in obstructive cholestasis. Hepatology.

[17] Hara Y., Sassi Y., Guibert C., Gambaryan N., Dorfmuller P., Eddahibi S., Lompre A.M., Humbert M., Hulot J.S. (2011). Inhibition of MRP4 prevents and reverses pulmonary hypertension in mice. J. Clin. Investig..

[18] Matsumiya W., Kusuhara S., Hayashibe K., Maruyama K., Kusuhara H., Tagami M., Schuetz J.D., Negi A. (2012). Forskolin modifies retinal vascular development in Mrp4-knockout mice. Investig. Ophthalmol. Vis. Sci..

[19] Sassi Y., Lipskaia L., Vandecasteele G., Nikolaev V.O., Hatem S.N., Cohen-Aubart F., Russel F.G., Mougenot N., Vrignaud C., Lechat P. (2008). Multidrug resistance-associated protein 4 regulates cAMP-dependent signaling pathways and controls human and rat SMC proliferation. J. Clin. Investig..

[20] Fu Z.D., Csanaky I.L., Klaassen C.D. (2012). Effects of aging on mRNA profiles for drug-metabolizing enzymes and transporters in livers of male and female mice. Drug Metab. Dispos..

[21] Carillion A., Feldman S., Jiang C., Atassi F., Na N., Mougenot N., Besse S., Hulot J.S., Riou B., Amour J. (2015). Overexpression of cyclic adenosine monophosphate effluent protein MRP4 induces an altered response to beta-adrenergic stimulation in the senescent rat heart. Anesthesiology.

[22] Huang H., Lu-Bo Y., Haddad G.G. (2014). A Drosophila ABC transporter regulates lifespan. PLoS Genet..

[23] Berthier J., Arnion H., Saint-Marcoux F., Picard N. (2019). Multidrug resistance-associated protein 4 in pharmacology: Overview of its contribution to pharmacokinetics, pharmacodynamics and pharmacogenetics. Life Sci..

[24] Cheung L., Yu D.M., Neiron Z., Failes T.W., Arndt G.M., Fletcher J.I. (2015). Identification of new MRP4 inhibitors from a library of FDA approved drugs using a high-throughput bioluminescence screen. Biochem. Pharmacol..

[25] Tachikawa M., Toki H., Tomi M., Hosoya K. (2008). Gene expression profiles of ATP-binding cassette transporter A and C subfamilies in mouse retinal vascular endothelial cells. Microvasc. Res..

[26] Tagami M., Kusuhara S., Honda S., Tsukahara Y., Negi A. (2009). Expression of ATP-binding cassette transporters at the inner blood-retinal barrier in a neonatal mouse model of oxygen-induced retinopathy. Brain Res..

[27] Katsuyama A., Kusuhara S., Asahara S.I., Nakai S.I., Mori S., Matsumiya W., Miki A., Kurimoto T., Imai H., Kido Y. (2020). En face slab optical coherence tomography imaging successfully monitors progressive degenerative changes in the innermost layer of the diabetic retina. BMJ Open Diabetes Res. Care.

[28] Zudaire E., Gambardella L., Kurcz C., Vermeren S. (2011). A computational tool for quantitative analysis of vascular networks. PLoS ONE.

[29] Mori S., Kurimoto T., Miki A., Maeda H., Kusuhara S., Nakamura M. (2020). Aqp9 Gene Deletion Enhances Retinal Ganglion Cell (RGC) Death and Dysfunction Induced by Optic Nerve Crush: Evidence that Aquaporin 9 Acts as an Astrocyte-to-Neuron Lactate Shuttle in Concert with Monocarboxylate Transporters To Support RGC Function and Survival. Mol. Neurobiol..

[30] Moshiri A., Gonzalez E., Tagawa K., Maeda H., Wang M., Frishman L.J., Wang S.W. (2008). Near complete loss of retinal ganglion cells in the math5/brn3b double knockout elicits severe reductions of other cell types during retinal development. Dev. Biol..

[31] Kusuhara S., Fukushima Y., Ogura S., Inoue N., Uemura A. (2018). Pathophysiology of Diabetic Retinopathy: The Old and the New. Diabetes Metab. J..

[32] Ogura S., Kurata K., Hattori Y., Takase H., Ishiguro-Oonuma T., Hwang Y., Ahn S., Park I., Ikeda W., Kusuhara S. (2017). Sustained inflammation after pericyte depletion induces irreversible blood-retina barrier breakdown. JCI Insight.

[33] Kusuhara S., Fukushima Y., Fukuhara S., Jakt L.M., Okada M., Shimizu Y., Hata M., Nishida K., Negi A., Hirashima M. (2012). Arhgef15 promotes retinal angiogenesis by mediating VEGF-induced Cdc42 activation and potentiating RhoJ inactivation in endothelial cells. PLoS ONE.

[34] An M.J., Kim C.H., Nam G.Y., Kim D.H., Rhee S., Cho S.J., Kim J.W. (2018). Transcriptome analysis for UVB-induced phototoxicity in mouse retina. Environ. Toxicol..

[35] Qiu Y., Yu P., Lin R., Fu X., Hao B., Lei B. (2017). Genome-wide retinal transcriptome analysis of endotoxin-induced uveitis in mice with next-generation sequencing. Mol. Vis..

[36] Wang J., Geisert E.E., Struebing F.L. (2019). RNA sequencing profiling of the retina in C57BL/6J and DBA/2J mice: Enhancing the retinal microarray data sets from GeneNetwork. Mol. Vis..

[37] Kase S., He S., Sonoda S., Kitamura M., Spee C., Wawrousek E., Ryan S.J., Kannan R., Hinton D.R. (2010). alphaB-crystallin regulation of angiogenesis by modulation of VEGF. Blood.

[38] Zhang Y., Schuetz J.D., Elmquist W.F., Miller D.W. (2004). Plasma membrane localization of multidrug resistance-associated protein homologs in brain capillary endothelial cells. J. Pharmacol. Exp. Ther..

[39] Liu Y.T., Liu W., Zhu G.Y., Wang F.L., Chen Q. (2018). Involvement of multidrug resistance protein 4 in the hepatocyte efflux of lamivudine and entecavir. Mol. Med. Rep..

[40] Nishiguchi K.M., Carvalho L.S., Rizzi M., Powell K., Holthaus S.M., Azam S.A., Duran Y., Ribeiro J., Luhmann U.F., Bainbridge J.W. (2015). Gene therapy restores vision in rd1 mice after removal of a confounding mutation in Gpr179. Nat. Commun..

[41] Donato L., Scimone C., Alibrandi S., Pitruzzella A., Scalia F., D’Angelo R., Sidoti A. (2020). Possible A2E Mutagenic Effects on RPE Mitochondrial DNA from Innovative RNA-Seq Bioinformatics Pipeline. Antioxidants.

[42] Donato L., Scimone C., Alibrandi S., Rinaldi C., Sidoti A., D’Angelo R. (2020). Transcriptome Analyses of lncRNAs in A2E-Stressed Retinal Epithelial Cells Unveil Advanced Links between Metabolic Impairments Related to Oxidative Stress and Retinitis Pigmentosa. Antioxidants.

[43] Scimone C., Alibrandi S., Scalinci S.Z., Trovato-Battagliola E., D’Angelo R., Sidoti A., Donato L. (2020). Expression of Pro-Angiogenic Markers Is Enhanced by Blue Light in Human RPE Cells. Antioxidants.

