# Supplementary material for: Changes in Gene Expression Profiling and Phenotype in Aged Multidrug Resistance Protein 4-Deficient Mouse Retinas
Source: Antioxidants (Basel). 2021 Mar 15;10(3):455. doi: 10.3390/antiox10030455 (PMC7999859; doi:10.3390/antiox10030455)
Supplement: Supplementary file 1 [file antioxidants-10-00455-s001.pdf]

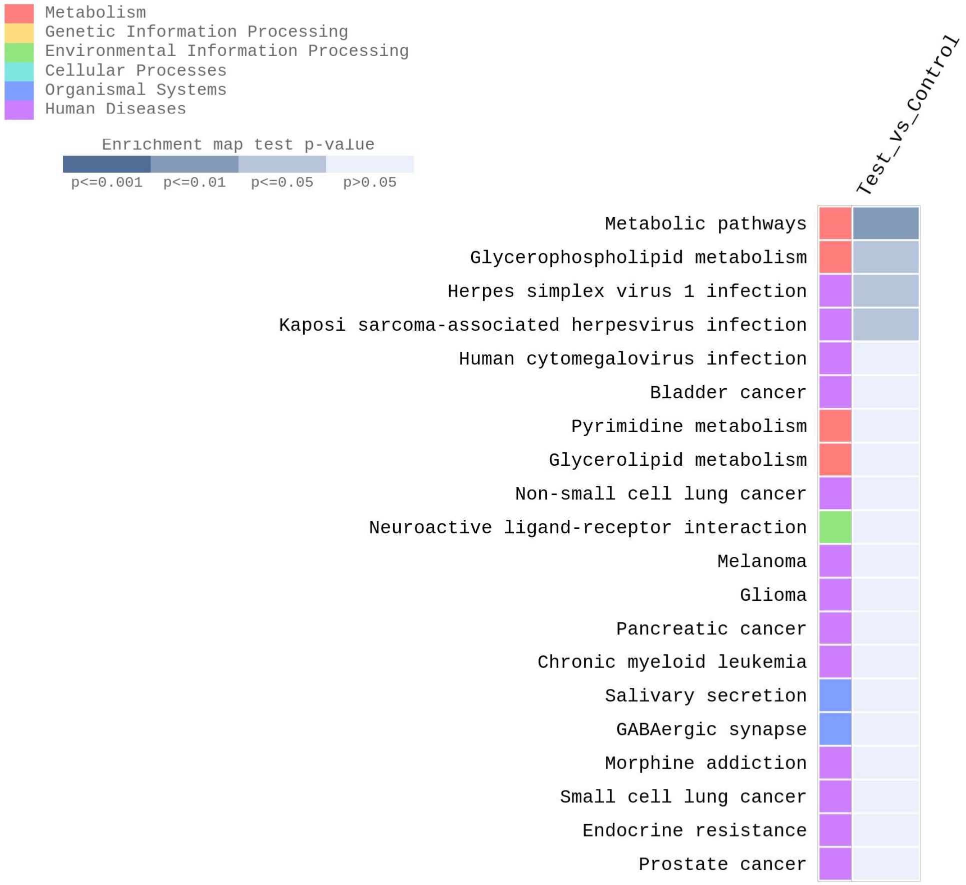

Figure S1. KEGG pathway analysis result. Test, *Mrp4*-null mice; Control, wild type mice.

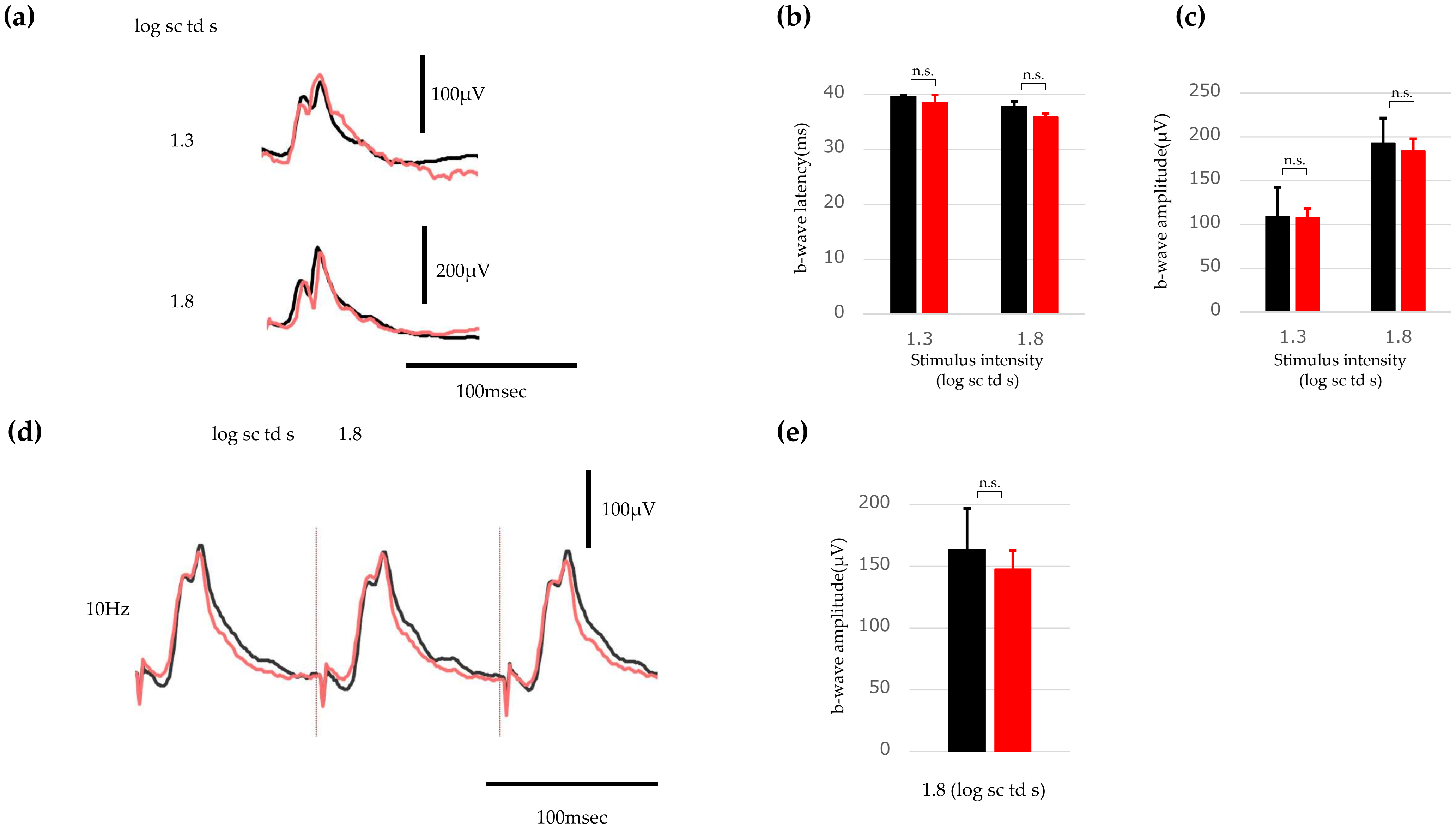

Figure S2. Results of photopic electroretinogram (ERG) phenotype. (a) Representative light-adapted ERG recordings. **(b)** Quantitative analyses of b-wave latency (n=6). **(c)** Quantitative analyses of b-wave amplitude (n=6). **(d)** Representative light-adapted flicker ERG recordings. **(e)** Quantitative analyses of b-wave amplitude of flicker ERG. Error bar = standard deviation. Black line or black bars are data from aged (45 weeks old) WT mice and red line or red bars are data from aged (45 weeks old) Mrp4-null mice. n.s., not significant.

Table S1. Differentially expressed gene list

ProbeID	Gene_Symbol	Gene_ID	RefSeqAccess	Test/Control	Test/Control.	Test/Control.	Test/Control.l	Test/Control.	Control.mean	Test.mean	Control.sd	Test.sd
A_52_P63086	Abcc4	239273	NM_0010333	15.131763	8.566687	0	18.39809872	0.306577	6.828236	10.747744	0.293362	0.089042
A_51_P43178	Myom2	17930	NM_008664	13.353321	6.512161	0	14.20811725	0.361416	4.905649	8.644776	0.294783	0.209104
A_51_P15112	Cd52	23833	NM_013706	7.874139	9.170686	0	14.5468379	0.232608	7.802149	10.779272	0.173422	0.155020
A_55_P21855	Masp2	17175	NM_010767	4.247988	6.581830	3.4375E-12	7.926643592	0.303288	5.620629	7.707409	0.198494	0.229312
A_51_P36800	E2f2	242705	NM_177733	3.894574	6.726813	6.0088E-12	7.847449417	0.299743	5.817197	7.778662	0.142175	0.263879
A_51_P44515	Spry2	24064	NM_011897	3.755293	6.174973	5.1567E-10	7.219594839	0.271622	5.293840	7.202765	0.195367	0.188707
A_30_P01028				3.664921	7.284358	2.5157E-12	7.977896654	0.488237	6.407470	8.281252	0.161421	0.460781
A_51_P25140	Tgds	76355	NM_029578	3.344442	8.115233	1.1996E-11	7.740803517	0.857588	7.290946	9.032712	0.655251	0.553267
A_51_P11296	Ch25h	12642	NM_009890	3.289766	5.427424	0.00744576	4.160288043	1.276850	4.635987	6.353972	0.515972	1.167955
A_30_P01033				3.262382	7.941787	4.2497E-11	7.56712464	0.128015	7.134497	8.840423	0.052669	0.116679
A_55_P25657	Gm33272	102636107	XR_00178292	3.245421	11.137295	7.2982E-12	7.819635275	0.964553	10.320421	12.018826	0.655385	0.707696
A_55_P29038	1700123L14R	78482	NR_003643	3.064911	5.710272	1.0561E-05	5.591267836	0.355828	4.959221	6.575066	0.278199	0.221853
A_51_P31169	Serpine3	319433	NM_0011999	3.032855	7.805183	1.0407E-09	7.116053357	0.837163	7.045771	8.646447	0.565601	0.617201
A_51_P44572	Pirb	18733	NM_011095	2.903616	5.029974	0.01834586	3.918409754	1.489531	4.319481	5.857332	0.347333	1.448469
A_66_P13787	A930038B10R	320323	XR_383352	2.899178	8.362446	3.3749E-10	7.280832235	0.312435	7.629800	9.165444	0.144953	0.276774
A_55_P21809	Optc	269120	NM_054076	2.882836	9.237883	1.1012E-11	7.756983258	0.804022	8.505657	10.033145	0.535482	0.599759
A_55_P25657	Gm33272	102636107	XR_00178292	2.871745	11.185010	5.9542E-09	6.847314003	0.929518	10.449902	11.971829	0.673861	0.640246
A_55_P25657	Gm33272	102636107	XR_00178292	2.862137	10.719443	7.1653E-09	6.817444587	0.935216	9.987702	11.504795	0.613403	0.705951
A_66_P13839	Cd101	630146	NM_0011679	2.840955	5.788788	0.00032816	4.890740796	0.379058	5.084394	6.590770	0.217540	0.310421
A_55_P21379				2.761933	7.629550	2.3315E-08	6.630772701	1.581876	6.931826	8.397504	1.484144	0.547400
A_55_P19719	Kdf1	69073	NM_0010839	2.756962	7.030321	7.676E-06	5.653328624	0.245497	6.336739	7.799818	0.196695	0.146902
A_55_P22519	A930038B10R	320323	XR_383352	2.747192	8.170352	1.8756E-09	7.017113196	0.398426	7.473829	8.931787	0.128053	0.377288
A_55_P21573	Gm5741	100503710	NM_0011955	2.706046	5.985004	9.75E-05	5.146346537	0.387650	5.309836	6.746023	0.360587	0.142301
A_55_P19701	Ltbp2	16997	XM_0065155	2.684494	7.991640	2.3153E-06	5.870052274	0.698596	7.310999	8.735649	0.378129	0.587414
A_66_P10225	Agpat5	52123		2.679310	7.071660	1.3447E-06	5.96697756	0.322016	6.396375	7.818236	0.046320	0.318667
A_55_P19574	Ggnbp1	70772	NM_0012518	2.671163	6.025861	1.2861E-05	5.554683326	0.337109	5.358663	6.776131	0.305394	0.142747
A_55_P21673	Cort	12854	NM_007745	2.618786	7.589678	2.4568E-07	6.26224261	0.347887	6.926933	8.315831	0.286394	0.197494
A_52_P58848	Fbln1	14114	NM_010180	2.459456	7.370563	0.00320994	4.36041732	0.772011	6.749927	8.048266	0.538738	0.552959
A_55_P27403	Fbln1	14114		2.457980	8.225316	7.6235E-05	5.198174972	0.731293	7.602123	8.899596	0.269512	0.679818
A_55_P19544	Gm10475	100038682		2.418602	5.236481	0.00056013	4.768476392	0.801209	4.638007	5.912180	0.377127	0.706902
A_51_P40068				2.418113	7.957492	3.5332E-05	5.357583596	0.384870	7.346002	8.619883	0.223257	0.313498
A_55_P27235	Zfp600	667666	NM_0011775	2.398979	6.681323	0.00017888	5.017873506	0.275363	6.079863	7.342283	0.230403	0.150796
A_52_P35678	Pdzd8	107368	NM_0010332	2.398265	9.920150	7.0704E-08	6.459224855	0.140524	9.309202	10.571193	0.096926	0.101746
A_55_P20798	Asap3	230837	NM_0013556	2.387573	9.334899	9.2686E-07	6.032555347	0.154795	8.728212	9.983757	0.090283	0.125739
A_66_P13788	Gm39121	105243109	XR_870092	2.378229	9.102550	1.0459E-06	6.010416179	0.129082	8.499034	9.748922	0.046006	0.120605
A_51_P36537	Best2	212989	NM_0011301	2.357293	7.533816	0.00071163	4.713224917	0.675469	6.940602	8.177733	0.115735	0.665480
A_65_P15571	Mid1	17318		2.274001	9.088106	2.2981E-06	5.87377883	0.536528	8.514791	9.700024	0.499704	0.195342
A_51_P13688	Rb1	19645	NM_009029	2.273273	10.931696	8.8245E-08	6.422756208	0.200764	10.355350	11.540121	0.097265	0.175630
A_51_P14259	Rnf207	433809	NM_0010334	2.265418	7.901949	6.9266E-05	5.21995497	0.837816	7.334047	8.513825	0.594208	0.590637
A_51_P51431	Slc13a4	243755	NM_172892	2.261694	8.698561	0.00012053	5.096893974	0.540527	8.129758	9.307161	0.311502	0.441741
A_52_P48054	Tnpo2	212999	NM_145390	2.247562	9.955688	8.792E-07	6.043699686	0.352245	9.388632	10.556993	0.155626	0.316002
A_55_P27417	Dio3os	353504	NR_002866	2.234988	7.685615	0.00058443	4.756516917	0.552539	7.127346	8.287613	0.523836	0.175772
A_30_P01031				2.221082	12.863797	5.0074E-11	7.541586237	0.401339	12.301039	13.452301	0.267175	0.299484
A_51_P18169	Nod2	257632	NM_145857	2.183147	7.652883	0.00273181	4.398615043	0.341563	7.110375	8.236784	0.219912	0.261351
A_65_P08768	Fbln1	14114	NM_0013470	2.167039	6.857159	0.00541894	4.238281111	0.703067	6.321952	7.437677	0.307867	0.632077
A_30_P01018				2.111589	5.944133	0.02066611	3.886812717	0.192126	5.429371	6.507700	0.146591	0.124192
A_66_P11500	4930528J11Ri	75224		2.088147	7.085643	0.00192908	4.476580818	0.278334	6.574409	7.636632	0.171150	0.219493
A_55_P24356	Xpo7	65246		2.029477	8.928203	0.00164403	4.515207083	0.264470	8.432235	9.453342	0.247337	0.093640
A_52_P32397	Farsa	66590		2.015847	9.211624	4.4685E-05	5.308657927	0.292992	8.719801	9.731187	0.264400	0.126241
A_55_P20167	Slc30a2	230810	NM_0010396	1.992247	6.419073	0.00777245	4.148969601	0.348591	5.941102	6.935498	0.261685	0.230298
A_51_P11666	2610005L07R	381598		1.985771	12.038139	7.8414E-06	5.647324745	0.156512	11.553456	12.543155	0.146038	0.056293
A_52_P51557	Slc18a2	214084	NM_172523	1.970303	9.491843	0.00010834	5.124600843	0.310090	9.015233	9.993650	0.174260	0.256494
A_51_P43153	Prss56	69453	NM_027084	1.956231	11.678468	0.00127362	4.570545007	0.481483	11.204456	12.172533	0.270964	0.398000
A_52_P33617	Gabra2	14395	NM_008066	1.949835	8.475410	0.00084908	4.673793192	0.607368	8.007410	8.970763	0.323563	0.514007
A_51_P22345	Polr3d	67065	NM_025945	1.940470	8.764705	0.0011864	4.590876909	0.268269	8.299538	9.255944	0.236461	0.126705
A_55_P27315	Gabra2	14395	NM_008066	1.924890	8.296082	0.00654245	4.192735536	0.245503	7.837132	8.781909	0.153643	0.191483
A_51_P28967	Npm2	328440	NM_181345	1.924213	6.038752	0.02974579	3.77964258	0.285417	5.585046	6.529315	0.233065	0.164753
A_52_P56989	5430419D17R	71395	XM_0065084	1.915721	10.639770	0.00054592	4.775362507	0.316141	10.181155	11.119043	0.211026	0.235401
A_55_P21377	Zfp981	100041433	NM_0012431	1.914302	7.467507	0.01143262	4.043510542	0.190501	7.013774	7.950592	0.105798	0.158422
A_55_P27148	Zfp600	667666	NM_0011775	1.913890	7.484987	0.0218677	3.868923331	0.165205	7.031366	7.967873	0.107875	0.125123
A_51_P29200	Gpx3	14778	NM_008161	1.902455	8.800253	0.00124565	4.578297475	0.612742	8.348542	9.276405	0.293784	0.537720
A_55_P27158	Zfp600	667666	NM_0011775	1.895843	7.598935	0.01623696	3.951938096	0.160369	7.151511	8.074350	0.089233	0.133250
A_51_P21908	Alpk2	225638	NM_0010372	1.889365	7.427187	0.02420467	3.839990839	0.226654	6.982402	7.900304	0.040035	0.223090
A_51_P35696	Lgi3	213469	NM_145219	1.883136	8.785428	0.00808608	4.138101141	0.337267	8.340715	9.253852	0.122478	0.314243
A_52_P68314	Cdh11	12552	NM_009866	1.871767	11.747644	0.00011559	5.110459491	0.454504	11.304144	12.208545	0.328094	0.314528
A_55_P28474	Gm9909	102634904	XR_388230	1.868900	9.640951	0.00094938	4.645888743	0.187705	9.200404	10.102593	0.150738	0.111854
A_30_P01033				1.863139	9.211665	0.01108573	4.052156334	0.288455	8.773727	9.671462	0.247982	0.147349
A_55_P23143	BC065403	100502819		1.841331	7.410166	0.01506296	3.97239217	0.534581	6.982866	7.863615	0.394846	0.360380
A_66_P11329	Erdr1	170942	NM_133362	1.823800	15.460545	4.6789E-06	5.741780223	0.317560	15.033147	15.900094	0.275451	0.158024
A_65_P14895				1.818596	11.004072	0.00115977	4.597941918	0.251078	10.581113	11.443937	0.186668	0.167915
A_30_P01030				1.808982	8.768632	0.03813581	3.714186364	0.651567	8.351462	9.206640	0.463547	0.457891
A_55_P20427	Cltrn	57394	NM_020626	1.808053	8.385983	0.00058443	4.757502827	0.453593	7.969640	8.824077	0.160378	0.424295
A_55_P20279	Gabra2	14395	NM_008066	1.802817	9.316584	0.00912602	4.103177497	0.176027	8.901152	9.751405	0.125755	0.123171
A_55_P28095	Polr3d	67065	NM_025945	1.800836	9.607935	0.01101333	4.055127415	0.168597	9.192968	10.041634	0.129041	0.108506
A_55_P20121				1.788061	9.210868	0.0242589	3.83808839	0.380158	8.801204	9.639600	0.235804	0.298189
A_30_P01028				1.787694	13.849329	1.6138E-05	5.512631927	0.156994	13.436617	14.274717	0.092293	0.127000
A_66_P11041	Cdh11	12552	NM_009866	1.786810	9.630934	0.01348175	4.001839286	0.383568	9.221338	10.058724	0.252831	0.288445
A_51_P10298	Penk	18619	NM_0010029	1.785283	11.522581	0.000181	5.013769353	0.574714	11.112086	11.948239	0.295884	0.492695

A_55_P21773	Gabra2	14395	NM_008066	1.779057	8.671417	0.02270925	3.858335848	0.177164	8.265812	9.096925	0.146573	0.099516
A_51_P29967	Rcc2	108911	NM_173867	1.766487	11.659702	0.00329614	4.351592693	0.416138	11.256482	12.077365	0.302542	0.285726
A_55_P27372	2610005L07R	381598		1.741874	11.716476	0.00190663	4.480610984	0.200734	11.322993	12.123633	0.168924	0.108438
A_55_P23815				1.740124	12.414389	0.00050576	4.794175753	0.129328	12.021224	12.820414	0.051122	0.118795
A_55_P20775	Sod3	20657	NM_011435	1.727098	8.160330	0.02459984	3.833312115	0.413050	7.775670	8.564020	0.046105	0.410469
A_51_P14672	Col19a1	12823	NM_007733	1.702280	9.033059	0.02117855	3.878096054	0.566226	8.657472	9.424940	0.277391	0.493626
A_30_P01023				1.684089	12.065845	0.00562305	4.228455958	0.250979	11.695717	12.447685	0.136013	0.210928
A_30_P01032				1.661856	12.176321	0.01189432	4.032795057	0.184683	11.815435	12.548230	0.074239	0.169104
A_66_P10592	A930014D07R	77947		1.614649	10.201954	0.03813581	3.713311504	0.265491	9.862196	10.553417	0.112062	0.240682
A_52_P11441	Rab6b	270192	NM_173781	-1.504088	14.367004	0.01847899	-3.91527585	0.161857	14.664466	14.075576	0.098150	0.128702
A_55_P27389	Uchl1	22223	NM_011670	-1.587259	15.277065	1.7741E-05	-5.49369626	0.177941	15.613969	14.947431	0.117323	0.133784
A_55_P19762	Arf5	11844	NM_007480	-1.607013	11.980955	0.01683486	-3.94187182	0.136188	12.328032	11.643650	0.098947	0.093577
A_52_P53484	Atp8a1	11980	NM_00103899	-1.634513	10.088237	0.02405071	-3.84291658	0.221695	10.448891	9.740031	0.065581	0.211773
A_66_P10931	Snapc2	102209	NM_133968	-1.641959	11.666011	0.02889749	-3.7894987	0.229401	12.029203	11.313785	0.117417	0.197074
A_55_P22134	Hnrnpr	74326	NM_00135518	-1.701264	9.381071	0.04685161	-3.65691926	0.138605	9.772202	9.005595	0.067762	0.120912
A_52_P63852	Cfap74	544678		-1.708350	5.281622	0.04325036	-3.67869638	0.611389	5.682032	4.909429	0.488517	0.367623
A_55_P20461	Xlr4b	27083	NM_021365	-1.716846	5.663605	0.00899452	-4.1079905	0.959702	6.066889	5.287128	0.748699	0.600397
A_51_P16906	Lpcat2	270084	NM_173014	-1.718899	9.994920	0.02698249	-3.80917252	0.249756	10.393297	9.611812	0.179766	0.173384
A_55_P19925	Entpd4b	100862375	NM_00135915	-1.734968	12.706401	0.00053259	-4.78205993	0.165849	13.110070	12.315162	0.129327	0.103828
A_51_P43778	Rbm28	68272	NM_133925	-1.771541	11.654025	0.00072025	-4.70910908	0.308561	12.073826	11.248820	0.077377	0.298702
A_55_P20421	Bzw2	66912	XM_00651516	-1.781738	10.191593	0.00286975	-4.38638328	0.413204	10.616748	9.783463	0.117500	0.396145
A_30_P01025				-1.810399	8.841255	0.00468518	-4.2723505	2.624666	9.279770	8.423462	1.789752	1.919807
A_51_P31203	Cd300e	217306	NM_172050	-1.827356	6.975330	0.02897706	-3.78748313	0.547758	7.423752	6.553994	0.344647	0.425744
A_55_P20481				-1.849128	10.402192	0.00322907	-4.35760172	0.417589	10.855062	9.968217	0.110895	0.402595
A_55_P21265	Gm41515	105246181	XM_01124617	-1.899920	6.696148	0.00808608	-4.13692356	0.514965	7.175103	6.249164	0.324392	0.399948
A_55_P20278	Cryba4	12959	NM_021351	-1.903937	9.166090	0.0011864	-4.59004746	1.114579	9.642344	8.713359	0.821522	0.753251
A_51_P25565	2210011C24R	70134	NM_00129129	-1.905582	5.105615	0.01069268	-4.06347346	0.409287	5.591873	4.661641	0.229577	0.338837
A_55_P19897				-1.913945	10.522608	0.0026518	-4.40659469	0.434245	11.001297	10.064747	0.102489	0.421977
A_55_P27895	Scoc	56367	NM_019708	-1.916222	12.704511	5.9333E-05	-5.2505625	0.101267	13.182302	12.244038	0.014559	0.100215
A_51_P19562	Zfp330	30932	NM_145600	-1.926602	11.823733	8.8001E-05	-5.16944754	0.085036	12.306220	11.360162	0.080981	0.025947
A_55_P20602	Fam45a	67894	NM_00136062	-1.948633	12.336702	2.0443E-05	-5.46418438	0.329343	12.827315	11.864853	0.223296	0.242086
A_55_P28098	Lpcat2	270084	NM_173014	-1.959135	10.726656	0.00046414	-4.81487017	0.223748	11.222728	10.252511	0.145150	0.170278
A_55_P20788				-1.965414	12.081849	0.00040133	-4.84737056	0.298508	12.579094	11.604261	0.101607	0.280684
A_51_P35931	Apobec2	11811	NM_009694	-1.968541	7.639537	0.02078052	-3.88408744	0.448697	8.143707	7.166580	0.298034	0.335418
A_55_P28032	Gm13212	433801	NM_00132441	-1.970966	9.424656	0.00103203	-4.62539081	0.367329	9.926808	8.947905	0.076490	0.359277
A_51_P13406	Stxbp2	20911	NM_011503	-1.971100	9.181676	0.00176779	-4.49825582	0.191378	9.684215	8.705214	0.122038	0.147418
A_55_P22949	Ube4bos1	77822		-1.979083	10.357408	3.2475E-05	-5.37493122	0.434570	10.861523	9.876691	0.134125	0.413354
A_30_P01032				-1.981363	5.594188	0.0178878	-3.92589681	0.555924	6.109138	5.122645	0.405004	0.380819
A_51_P51440	Slc2a5	56485	NM_019741	-1.983219	5.620288	0.02858847	-3.79350699	0.194839	6.135872	5.148028	0.099563	0.167480
A_51_P30222	Entpd4	67464	NM_026174	-1.985247	10.015596	0.00049794	-4.79905025	0.133627	10.522463	9.533145	0.114130	0.069502
A_52_P66107	Snhg3	399101	NR_003270	-1.998644	11.281395	0.00014395	-5.06129889	0.335902	11.791959	10.792937	0.135733	0.307257
A_52_P38148	Spon2	100689	NM_133903	-2.001241	6.953732	0.01476874	-3.97879068	0.322074	7.472164	6.471269	0.252924	0.199402
A_52_P58290	Entpd4	67464	NM_026174	-2.068195	7.377554	0.00115977	-4.59857413	0.700732	7.920339	6.871966	0.276079	0.644054
A_55_P20674	Arf5	11844	NM_007480	-2.072794	12.074997	4.6789E-06	-5.74011783	0.296435	12.612227	11.560651	0.198364	0.220285
A_51_P34342	Slc25a37	67712	NM_026331	-2.079382	10.897681	8.727E-06	-5.6265775	0.238348	11.438546	10.382391	0.175152	0.161653
A_51_P14347	Timm44	21856	NM_011592	-2.081492	12.406417	3.8005E-07	-6.18803194	0.133882	12.946491	11.888873	0.125560	0.046466
A_52_P64379	Crybb1	12960	NM_023695	-2.132535	7.517248	0.00103203	-4.62562344	1.275217	8.083356	6.990787	0.941679	0.859895
A_55_P19848	Retn	57264	NM_00120495	-2.156617	8.968954	0.00011893	-5.10225626	0.211287	9.540457	8.431687	0.160505	0.137406
A_66_P10786	Crybb2	12961	NM_007773	-2.174201	8.001076	0.0012467	-4.57656468	1.312499	8.580909	7.460424	0.827634	1.018664
A_55_P24992	Timm44	21856	NM_011592	-2.185489	9.429368	7.391E-05	-5.20591762	0.150770	10.010197	8.882241	0.100139	0.112711
A_52_P37325	Atp11a	50770	NM_00129366	-2.186118	12.798833	3.8861E-08	-6.5520505	0.151360	13.375447	12.247076	0.041886	0.145449
A_55_P27356	Casz1	69743	NR_161310	-2.187635	12.123847	3.8005E-07	-6.18549997	0.433753	12.701676	11.572304	0.209850	0.379611
A_55_P27293	Cers4	67260	XM_00650886	-2.199642	8.966309	0.0003063	-4.90792916	0.361070	9.552957	8.415688	0.218830	0.287202
A_55_P20320	Uggt2	66435	NM_00108125	-2.222902	7.039512	0.00029895	-4.91453142	0.247489	7.639279	6.486834	0.194321	0.153264
A_55_P27177	Entpd4b	100862375		-2.243545	5.065012	0.01506296	-3.97126273	0.274660	5.681332	4.515552	0.135465	0.238929
A_52_P31835	Olfr1441	258678	NM_146683	-2.244227	7.200419	5.7096E-05	-5.25966871	0.503823	7.807100	6.640882	0.314402	0.393687
A_52_P96693				-2.257761	7.858112	0.00046414	-4.81597647	0.393601	8.467486	7.292593	0.226879	0.321633
A_55_P20634	Csmd1	94109	NM_053171	-2.261284	7.054071	2.7492E-05	-5.40701673	0.498809	7.667153	6.490011	0.377320	0.326252
A_51_P13407	Stxbp2	20911	NM_011503	-2.331445	5.239859	0.0345225	-3.74106303	0.572241	5.885929	4.664705	0.422170	0.386306
A_30_P01027				-2.346078	5.049701	0.00094938	-4.64738204	0.352032	5.702154	4.471903	0.150175	0.318393
A_52_P46566				-2.383941	7.977018	9.6963E-05	-5.14932757	0.427304	8.628270	7.374922	0.137443	0.404597
A_55_P20844				-2.401496	5.627906	0.00899452	-4.10829896	0.428453	6.295244	5.031310	0.300680	0.305228
A_52_P44871	Cacul1	78832		-2.427111	6.029808	0.0003389	-4.88259971	0.469069	6.703257	5.424018	0.308199	0.353609
A_55_P24285	Retn	57264	NM_00120495	-2.441919	9.000243	2.9829E-06	-5.82542788	0.268389	9.667262	8.379246	0.159747	0.215670
A_52_P68087	Ctxn1	330695	NM_183315	-2.442694	7.291472	0.00011893	-5.1012961	0.169005	7.964114	6.675641	0.164639	0.038165
A_51_P50285	Dgkh	380921		-2.456649	7.665145	3.6561E-05	-5.34927913	0.254125	8.340862	7.044170	0.180604	0.178779
A_30_P01032				-2.506047	4.640169	0.0003169	-4.89942327	0.358577	5.349961	4.024547	0.218009	0.284692
A_51_P45949	Crygs	12970	NM_009967	-2.536361	6.790423	3.4445E-06	-5.79890818	1.398874	7.494913	6.152153	1.025775	0.951123
A_51_P29880	Bfsp2	107993	NM_00100289	-2.594858	7.210714	3.6996E-05	-5.34504462	0.867061	7.931273	6.555617	0.717773	0.486412
A_66_P11361	Ube4bos1	77822		-2.596677	11.699039	3.9463E-12	-7.90590682	0.694906	12.407604	11.030938	0.196204	0.666632
A_51_P28529	Cryba4	12959	NM_021351	-2.604667	8.213553	4.8658E-07	-6.14369338	1.573550	8.933081	7.551982	1.040348	1.180566
A_52_P53008	Bfsp2	107993	NM_00100289	-2.614622	5.424484	5.6808E-05	-5.26265279	0.918984	6.161911	4.775309	0.805091	0.443124
A_55_P27282				-2.616088	7.029836	2.5723E-05	-5.42108334	0.215727	7.757686	6.370275	0.096264	0.193058
A_51_P30553	Eif2s3x	26905	NM_012010	-2.630929	9.058783	8.1728E-12	-7.79986651	1.047997	9.783404	8.387832	1.046981	0.046146
A_52_P88793	Zfp933	242747	NM_198619	-2.631468	9.516819	1.9999E-08	-6.65925954	0.590937	10.240311	8.844443	0.198320	0.556664
A_55_P20823	Crybb2	12961	NM_007773	-2.640709	9.559827	1.6705E-09	-7.03655713	1.788009	10.285917	8.884991	1.122813	1.391498
A_55_P20076	Cryaa	12954	NM_00127856	-2.717705	13.171758	4.4911E-16	-9.01228481	1.848967	13.912681	12.470293	1.122035	1.469598
A_52_P19237	Lgsn	266744	NM_153601	-2.718586	6.465686	1.6652E-09	-7.04035803	1.033404	7.227238	5.784381	0.905226	0.498486
A_55_P27454	Olfr1388	258459		-2.816555	8.035279	3.309E-07	-6.21282028	0.200014	8.816889	7.322958	0.188544	0.066757
A_55_P27237	Crnde	71296	NR_033641	-2.818503	7.147806	3.8982E-06	-5.77569669	0.266920	7.934247	6.439317	0.059181	0.260276
A_51_P11302	Cryba1	12957	NM_009965	-2.828611	9.584472	7.9539E-10	-7.15668639	2.051950	10.363822	8.863728	1.220200	1.649731

A_55_P21506	Gadd45gip1	102060	NM_183358	**-2.856331**	10.823646	**3.7104E-14**	-8.48900641	0.272733	11.607173	10.093010	0.186566	0.198939
A_55_P19752	Crygc	12966	NM_0010825	**-2.868233**	5.387395	**7.1653E-09**	-6.81451859	0.827928	6.200830	4.680668	0.817365	0.131828
A_55_P20039	Bfsp1	12075	NM_0012910	**-2.878969**	7.336527	**5.7199E-07**	-6.11528655	1.310896	8.138849	6.613297	1.057022	0.775342
A_51_P48013	Cryba2	12958	NM_021541	**-2.922684**	9.423721	**1.9028E-11**	-7.67481147	1.821295	10.229071	8.681777	1.125701	1.431751
A_52_P11103	Pcdh17	219228	NM_0010137	**-2.947979**	9.760810	**8.7573E-11**	-7.46449406	0.127527	10.571778	9.012052	0.106647	0.069924
A_55_P22343	Rnf150	330812	NM_177378	**-3.065135**	11.887784	**1.0964E-16**	-9.18000311	0.223726	12.723186	11.107235	0.073089	0.211451
A_55_P21149	Nek3	23954	NM_0011629	**-3.073766**	8.647729	**1.2293E-09**	-7.08944657	0.109198	9.495585	7.875578	0.050336	0.096904
A_66_P12793	Lim2	233187	NM_177693	**-3.095294**	7.038515	**1.5403E-13**	-8.31618202	1.939218	7.900585	6.270509	1.175980	1.541959
A_52_P59623	Fam204a	76539		**-3.231839**	4.686797	**2.2855E-06**	-5.87721902	0.262332	5.608749	3.916393	0.216776	0.147737
A_55_P21443	Gm4354	100043316		**-3.286575**	12.121234	**2.6248E-15**	-8.80343714	0.697197	13.009876	11.293291	0.249631	0.650975
A_52_P71541				**-3.370418**	5.521315	**1.3927E-08**	-6.715255	0.517298	6.466912	4.713985	0.337882	0.391705
A_55_P21699	Zfp982	195531	NM_0013654	**-3.444391**	7.579843	**3.4372E-09**	-6.92869572	0.574424	8.524287	6.740038	0.249805	0.517263
A_55_P20421				**-3.599972**	12.766711	**7.0302E-18**	-9.47902529	0.767963	13.724097	11.876112	0.239494	0.729664
A_55_P21257	Lrrc8e	72267	NM_028175	**-3.623833**	5.915894	**1.3433E-09**	-7.07365908	0.296208	6.917113	5.059597	0.106864	0.276259
A_51_P12826	Cyld	74256		**-3.864257**	6.305262	**1.3712E-11**	-7.72088168	0.327847	7.355310	5.405119	0.305786	0.118232
A_55_P19733	Gm3579	100041932		**-4.042077**	10.327412	**2.5686E-20**	-10.0644243	0.376212	11.383992	9.368896	0.083061	0.366928
A_55_P20752	Kbtbd11	74901	NM_029116	**-4.378432**	9.556372	**1.0906E-21**	-10.3798416	0.552336	10.680763	8.550348	0.236720	0.499038
A_55_P27379	Lgi3	213469		**-4.697855**	6.982873	**2.5326E-16**	-9.08190614	0.191003	8.187491	5.955489	0.113039	0.153962
A_52_P55560	Apbb2	11787		**-4.743147**	7.286215	**2.7815E-15**	-8.79086026	0.709104	8.495160	6.249315	0.184820	0.684595
A_30_P01031				**-4.905352**	7.035727	**9.3703E-16**	-8.92458521	0.192189	8.275816	5.981459	0.154316	0.114557
A_52_P52937	Enox1	239188	NM_172813	**-5.194814**	9.637597	**2.2943E-26**	-11.4068211	0.485835	10.899143	8.522071	0.215068	0.435639
A_66_P13143				**-5.677915**	6.300004	**3.043E-18**	-9.57429621	0.414846	7.676018	5.170657	0.312944	0.272330
A_52_P70381				**-5.933803**	7.266318	**1.0748E-21**	-10.3912863	0.182782	8.663453	6.094496	0.123948	0.134335
A_66_P13327				**-6.848760**	6.670604	**4.221E-25**	-11.1368744	0.294277	8.201385	5.425542	0.129655	0.264175
A_51_P36128	Agpat5	52123	NM_026792	**-7.499705**	7.703404	**1.3035E-29**	-12.0547685	0.152758	9.292731	6.385898	0.114676	0.100918
A_55_P20324	Slc25a37	67712	NM_026331	**-7.660266**	6.356264	**1.3575E-23**	-10.8110969	0.547651	7.992435	5.055041	0.316730	0.446770
A_55_P20036	4930480K23R	75016	NR_130157	**-10.853326**	7.131619	**9.4851E-41**	-14.027008	0.715109	9.056142	5.616076	0.430551	0.570970
